# Rapid and simultaneous determination of trigonelline, caffeine, and chlorogenic acid in green coffee bean extract

**DOI:** 10.1002/fsn3.2456

**Published:** 2021-07-05

**Authors:** Minaleshewa Atlabachew, Atakilt Abebe, Tessera Alemneh Wubieneh, Yilak Tefera Habtemariam

**Affiliations:** ^1^ Chemistry Department Science College Bahir Dar University Bahir Dar Ethiopia; ^2^ Material Science and Engineering Department Science College Bahir Dar University Bahir Dar Ethiopia

**Keywords:** caffeine, chlorogenic acid, green coffee, QuEChERS salt, salting‐out assisted liquid–liquid extraction, trigonelline, UV‐VIS

## Abstract

A simple, inexpensive, and rapid method for simultaneous determination of trigonelline, caffeine, and chlorogenic acid from green coffee bean extract was proposed based on salting‐out assisted liquid–liquid extraction, using QuEChERS salt and acetonitrile followed by UV‐Vis analysis. The proposed method represents acceptable linearity for trigonelline (0.9978), caffeine (0.9995), and chlorogenic acid (0.9996) with excellent correlation (0.93 and 0.83) for trigonelline and caffeine, respectively, when compared to RP‐HPLC‐DAD. The proposed method could be used in coffee industries for quality control and geographical origin traceability studies of green coffee samples.

## Graphical abstract

Salting‐out assisted liquid‐liquid extraction (SALLE) protocol is proposed for coffee alkaloids and chlorogenic acid extraction. An alternative UV‐VIS method is proposed for the simultaneous quantification of caffeine, chlorogenic acid, and trigonelline from green coffee extract

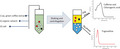

## 1 INTRODUCTION

Among the various agricultural outputs, coffee is one of the most popularly consumed beverage worldwide. Consequently, it has taken a significant market share in the World market and more than 121 countries are exporting coffee to different countries (http://www.ecea.org.et/uk/).

Analysis of caffeine, trigonelline, and chlorogenic acid levels in green coffee is vital for the coffee industry because they have a paramount effect on the overall quality of the brewed coffee and coffee products (de Maria et al., 1995). When coffee is roasted, most of the aroma and flavor are originated from trigonelline and chlorogenic acids (Heo et al., 2020; de Maria et al., 1995). Even though caffeine is unaffected during the roasting process, it is directly linked with its pharmacological effects (de Maria et al., 1995; Mehari, Redi‐Abshiro, Chandravanshi, Atlabachew, et al., 2016). Hence the quality of green coffee beans is directly related to the composition of these constituents (Mehari, Redi‐Abshiro, Chandravanshi, Atlabachew, et al., 2016; Mehari, Redi‐Abshiro, Chandravanshi, Combrinck, et al., 2016; Cheng et al., 2016).

The composition of these constituents varied significantly with coffee species, coffee varieties, and even in a coffee of the same variety but produced in different geographical locations (Mehari, Redi‐Abshiro, Chandravanshi, Atlabachew, et al., 2016; Mehari, Redi‐Abshiro, Chandravanshi, Combrinck, et al., 2016).

Several papers have been published for individual and simultaneous determination of two or three of these compounds from green coffee beans extract. A reversed‐phase HPLC‐with DAD has been reported for the simultaneous determination of caffeine and trigonelline (Casal et al., 1998; Mehari, Redi‐Abshiro, Chandravanshi, Atlabachew, et al., 2016) and chlorogenic acid (Vinson et al., 2019) analysis in green coffee bean extract. An alternative LC‐MS method was also reported for caffeine and trigonelline (Perrone et al., 2008) and chlorogenic acid (Mehari, Redi‐Abshiro, Chandravanshi, Combrinck, et al., 2016) analysis. A Gel filtration HPLC‐UV was reported for the simultaneous determination of caffeine, trigonelline, and chlorogenic acid in green coffee extract (De Maria et al., 1995). Although these methods were sensitive and accurate, they are expensive and need sample clean‐up techniques that lengthen the analysis time.

An alternative method based on UV‐Vis spectrometry was developed for the determination of caffeine (Belay et al., 2008) and chlorogenic acid (Dadoet al., 2019) in green coffee bean extracts. These methods are simple and inexpensive. However, they could not determine the other constituents simultaneously. Furthermore, repeated liquid–liquid extraction using dichloromethane was applied to exclude interfering compounds (trigonelline and chlorogenic acid).

Recently, UV‐Vis spectrometry methods were reported for a simultaneous determination of caffeine and chlorogenic acid with first‐order derivative spectra at 260 and 291 nm (Habtamu and Belay, 2020) and without derivative spectra at 272 and 330 nm (Navarra et al., 2017). Although these methods seem simple and fast, they are not selective and reliable. It has to be noted that green coffee bean contains a significant amount of trigonelline (up to 1.5% wt/wt) with a λmax at 264 nm. However, none of the methods has considered the presence of trigonelline in the extract and the elimination of its interference during caffeine determination.

Despite the range of relevant publications involving the state of the art analytical techniques for analysis of coffee secondary metabolites, there are still few studies that emphasize the use of modern sample preparation techniques that aim to minimize toxic solvents, time and extraction cost and removal of interferents from the matrix (Jeszka‐Skowron et al., 2015; Yılmaz and Kolak, 2017; De Azevedo et al., 2008; Theodoridis and Manesiotis, 2002; Syakfanaya et al., 2019).

The Quick, Easy, Cheap, Effective, Rugged, and Safe (QuEChERS) method, originally proposed for extraction of pesticides from fruits and vegetables (Galarce‐Bustos et al., 2019), is an extractive technique with excellent extraction performance, tunability according to the properties of the analytes, matrix constituents, and the final detection techniques (Galarce‐Bustos et al., 2019; Lehotay et al., 2010). The QuEChERS method involves the use of acetonitrile and salt mixture (MgSO4 and NaCl). Depending on the nature of the analytes and detection techniques, use of buffers and additional clean‐up techniques have been recommended (Galarce‐Bustos et al., 2019; Lehotay et al., 2010).

Although the QuEChERS method was widely applied for extraction of pesticides, herbicides, and other contaminants from various matrices, the modified QuEChERS method has also been applied for selective extraction of few natural products such as organic acids (Valente et al., 2017), alkaloids (Atlabachew et al., 2017), and flavonoids (Delgado‐Zamarreño et al., 2012) from natural product extracts have been described previously. However, this technique was not reported for caffeine, trigonelline, and chlorogenic acid from coffee. Furthermore, a simple, rapid, and accurate method for the determination of these compounds in green coffee is still a pressing demand for tracing the quality and authenticity of coffee beans since these compounds have been reported as a marker molecule to differentiate coffee varieties and coffee of different origin (Mehari, Redi‐Abshiro, Chandravanshi, Atlabachew, et al., 2016; Mehari, Redi‐Abshiro, Chandravanshi, Combrinck, et al., 2016; Ky et al., 2001; Bicho et al., 2013).

In this work, a simple, fast, and selective method based on a modified QuEChERS or salting‐out assisting liquid–liquid extraction followed by UV‐Vis the spectrometry method for the simultaneous determination of caffeine, chlorogenic acid, and trigonelline from green coffee beans is reported.

## 2 MATERIALS AND METHOD

### 2.1 Coffee samples

A total of three green coffee bean samples (300 g each) were collected from three sub‐districts of West Gojjam Zone of Amhara region, Ethiopia. About 50 g sample of green coffee beans was finely powdered using an electrical blender (FW100 high‐speed universal disintegrator), sieved using a 200‐µm‐ mesh size sieve, and stored in an airtight plastic bag.

### 2.2 Chemicals

All reagents used in this study were of analytical grade and directly used as received. Caffeine was purchased from Merck, trigonelline hydrochloride and 5‐caffeoylquinic acid were purchased from Sigma‐Aldrich, HPLC grade Acetonitrile and acetic acid were obtained from BDH, phosphoric acid (85%) was purchased from Tianjin Chemicals Int'l Co., Ltd, sodium chloride, magnesium sulfate anhydrous and lead acetate were purchased from Research Lab Fine Chem Industries, PVDF membrane syringe filter with 0.45 µm pore size and deionized water were used.

### 2.3 Instruments

Ultrasonic cleaner (Daihan Scientific), Agilent 1,260 series HPLC‐DAD (Agilent Technologies Inc., ), equipped with a quaternary pump and fitted with C8 column (Supelco, 15 cm × 4.6 mm, 5 μm, USA), Cary 60 UV‐Vis Spectrometer (Agilent technologies).

### 2.4 Extraction of the analytes from the coffee bean powder for UV‐VIS analysis

Exactly 0.200 g of sample was placed in a 50 ml Eppendorf tube and extracted with 10 ml of 1% aqueous acetic acid under ultrasonic water bath extractor at room temperature. After 90 min, the extract was centrifuged at 1,700 *g*, decanted, and the residue was reextracted with the same solvent. After centrifugation, the filtrates were combined filtered using a Nylon membrane syringe filter and kept in the refrigerator at 4°C until analysis.

### 2.5 Modified QuEChERS extraction

#### 2.5.1 Optimizing of extraction cycles

The zwitterion nature of trigonelline was helpful in a salting‐out assisted LLE extraction. Because; it was assumed that in acidic conditions, trigonelline prefers to stay in the aqueous phase while caffeine and chlorogenic acid partitions in the acetonitrile phase. Hence, before applying this assumption to the coffee extract, the procedure was optimized using a standard solution of each analyte. Briefly, 4 ml of 50 ppm caffeine and chlorogenic acid and 40 ppm trigonelline dissolved in 1%, 2%, and 3% acetic acid were taken in 15 ml Eppendorf tube and mixed with 4 ml of acetonitrile. Then, to each tube, 0.5 g of NaCl and 1.0 g of anhydrous MgSO_4_ was added. The mixture was shaken vigorously and centrifuged. The upper phase (acetonitrile phase) from each tube was taken with a micropipette and kept for UV‐Vis analysis. A second and a third extraction phase was applied on the aqueous phase by adding another portion of successive 4 ml of acetonitrile repeating the above processes.

The fractions in each phase (1st, 2nd, and 3rd) were analyzed by UV‐VIS spectrometer. Similarly, 4 ml of stock solution (untreated solution) of each standard solution was also analyzed as a reference solution.

Besides, the same procedure was applied to the coffee sample extract. Namely, 50 μL of the above coffee sample extract was diluted to 4 ml with 1% aqueous acetic acid. Extractions in three phases were carried out similarly to the above standard solution. For comparison, the same 50 μl was diluted to 4 ml and directly analyzed by UV‐VIS.

#### 2.5.2 Quantification of caffeine, trigonelline, and chlorogenic acid using UV‐VIS

During the optimization step, it was found that both caffeine and chlorogenic acid were completely partitioned into the acetonitrile phase in two extraction cycles while trigonelline remained in the aqueous phase. Based on this, the first two extracts were combined and analyzed by UV‐VIS for the quantification of caffeine and chlorogenic acid. The absorbance of trigonelline (Equation 1) was obtained at 264 nm from the difference in absorbance before and after QuEChERS salt extraction.

(1) $$A_{\text{trigonolline at 264 nm}}=A_{\text{reference solution or crude extract at 264 nm}}‐A_{\text{QuEChERS extract at 264 nm}}$$

Thus, concentrations of trigonelline in coffee samples were determined from the calibration curve constructed by plotting absorbance versus concentration (0.4–40.0 mg/L). For caffeine and chlorogenic acid, equal concentrations of each analyte (0.5–50 mg/L) were mixed and directly analyzed. Chlorogenic acid was quantified from the absorbance at 325 nm in the graph plotted as a function of chlorogenic acid concentration, since caffeine could not absorb beyond 310 nm. For caffeine, even if its λmax is 274 nm, chlorogenic acid also absorbs in this region. Thus, absorbance of caffeine was obtained by subtracting the absorbance of chlorogenic acid at 274 nm from the total absorbance at 274 (Equation 2). But, it was found that the absorbance of chlorogenic acid at 274 nm is about 0.37 times its absorbance at 325 nm. This was determined experimentally by analyzing the different concentration of chlorogenic acid standards. Therefore, in the mixture of caffeine and chlorogenic acid, the absorbance of caffeine (A_caff_ ) was taken as (Equation 2):

(2) $$A_{\text{caff}}=A_{274\;\text{nm}}‐0.37A_{325\;\text{nm}}$$

A calibration curve for caffeine was constructed by plotting the concentration of caffeine versus A_caff_ (the difference in absorbance obtained using the aforementioned formula).

### 2.6 Limit of detection (LOD) and limit of quantitation (LOQ)

The LOD and LOQ of Caffeine, chlorogenic acid, and trigonelline of the optimized methods were calculated using the calibration curve parameters following the method reported by Chopra et al., 2007. Accordingly, 3.3*σ*/*S* and 10*σ*/*S* were used to calculate LOD and LOQ, respectively, where S is slope and *σ* = standard error of the intercept of the calibration curve equation.

### 2.7 HPLC analysis of caffeine and trigonelline from coffee samples

To validate the developed extraction and analysis method, the coffee samples were extracted and analyzed by the HPLC‐DAD method reported by Mehari, Redi‐Abshiro, Chandravanshi, Combrinck, et al., 2016. A 0.4 g of green coffee powder was extracted with 60% methanol under an ultrasonic bath and a portion of the extract was treated with 20% lead acetate. The supernatant was filtered with a nylon membrane syringe filter and submitted to HPLC analysis. A quantity of 5.0 μl of the sample was injected into the column maintained at 25°C. The separation was carried out under isocratic conditions using 90% of acidified water (0.1% orthophosphoric acid) and 10 percent acetonitrile at a flow rate of 0.4 ml/min. Detection was made at 272 nm for both caffeine and trigonelline.

## 3 RESULTS AND DISCUSSION

Currently, a gel filtration HPLC‐UV method is being used for the accurate and simultaneous determination of trigonelline, caffeine, and chlorogenic acid from the green coffee extract. But, it is highly desirable to come up with a method that is simpler, faster, more reliable, more sensitive, and more economical. UV‐VIS methods are convenient for routine analysis of chlorogenic acid, caffeine, and trigonelline from green coffee extract for quality control and geographical origin traceability application.

**FIGURE 1:**
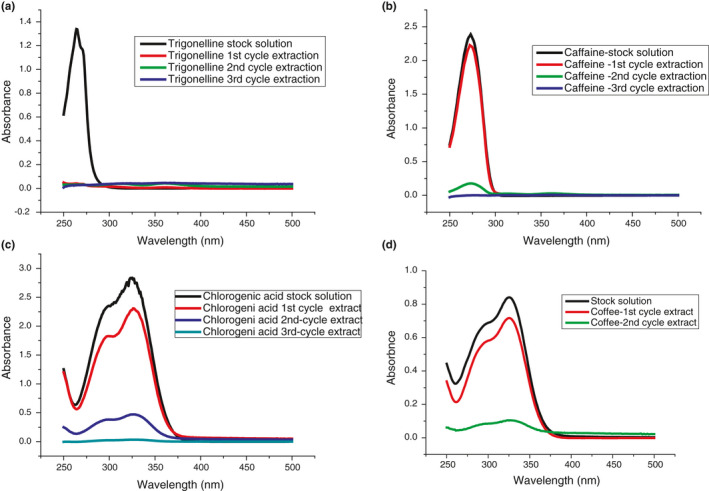
(a–d) Extraction cycle for (a) trigonelline, (b) caffeine, (c) chlorogenic acid, and (d) coffee sample

Based on this, the present study demonstrated the extended use of QuEChERS salt and acetonitrile for extraction and UV‐VIS determination of trigonelline, caffeine, and chlorogenic acid from green coffee bean extracts. During extraction, trigonelline was forced to remain in the aqueous phase (Figure 1a) while the latter two analytes were partitioned into the acetonitrile phase. Caffeine and chlorogenic acid were directly determined from the acetonitrile phase extract using the above equation (Equation 2). But trigonelline was determined by comparing the absorbance of the solution at 264 nm before and after QuEChERS extraction was carried out (Equation 1).

### 3.1 Optimization of the extraction cycle

In this study, extraction in three phases (or steps) using a medium prepared from an equal volume of acetonitrile and water was done. The caffeine (Figure 1b) and chlorogenic acid (Figure 1c) were effectively extracted in the acetonitrile phase, while trigonelline remained all the time in the aqueous phase (Figure 1a) A complete partitioning of caffeine and chlorogenic acid in just two extraction phases into the acetonitrile phase was noted.. Furthermore, while the first cycle extraction showed 81% of chlorogenic acid and 94% of caffeine transfer, 17% and 7% of chlorogenic acid and caffeine, respectively, were extracted during the 2nd cycle. Hence, it was concluded that a second extraction is sufficient to completely partition the two analytes into the acetonitrile phase.

The same extraction cycles were tested for green coffee sample extract and no recognizable absorbance values were noted between 250 and 500 nm after the second extraction cycle. Therefore, two extraction steps were sufficient to extract the analytes of interest in the acetonitrile phase (Figure 1d). The absorbance value of each analyte is indicated in Table 1.

**TABLE 1:** Analyzed absorbance data for Figure 1d spectra

Type of solution	UV‐VIS absorbance data			Absorbance values of each analyte		
	A_at 325 nm_	A_at 274 nm_	A_at 264_	Caffeine	Trigonelline	Chlorogenic acid
				A_at 274_–0.37A_325_	A_stock solution at 264 nm_–A_sum at 264 nm_	A_at 325_
Stock solution	0.841	0.457	0.338	0.395–0.37 (0.815) = 0.10975	0.338–0.265 = 0.0729	0.815
1st cycle extract	0.712	0.342	0.221			
2nd cycle extract	0.103	0.053	0.0441			
Sum of extracts (1stand 2ndext)	0.815	0.395	0.2651			

The effect of acid ratio (1%, 2%, and 3% v/v) on the partitioning of the three analytes into the acetonitrile phase was tested. Almost comparable extraction efficiency was observed for the three acid ratios. Therefore, 1% acetic acid in water was used to aid the partitioning of caffeine and chlorogenic acid into the acetonitrile phase while leaving trigonelline in the aqueous phase.

### 3.2 The calibration curve, LOD, and LOQ

**FIGURE 2:**
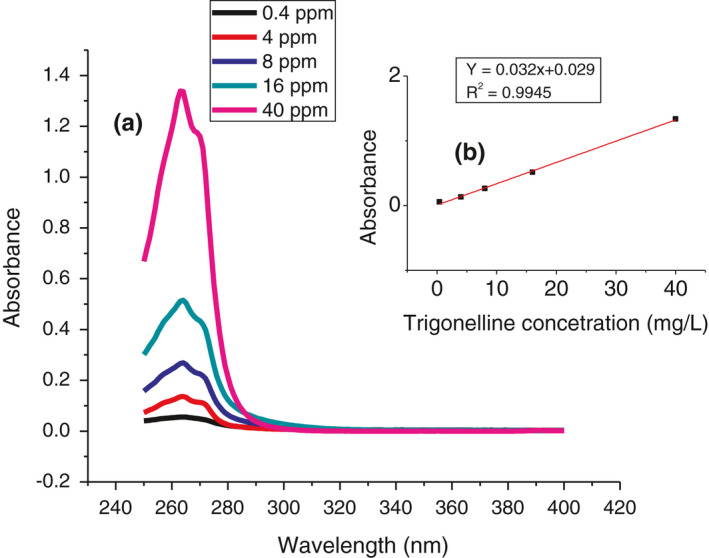
UV‐Vis spectra of trigonelline (a) and its calibration curve (b)

**FIGURE 3:**
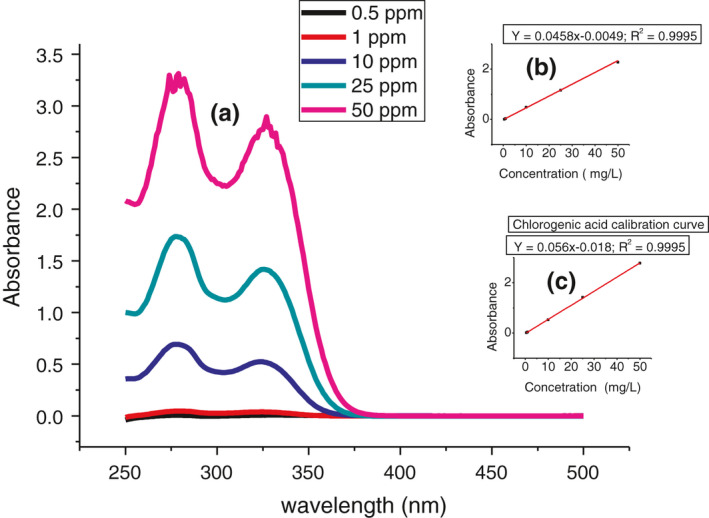
UV‐Vis spectra of caffeine and chlorogenic acid mixture (a); calibration curve of caffeine (b) and Chlorogenic acid (c). NB: Absorbance of caffeine was calculated using Equation 2

For the three analytes, the method was linear in the concentration range of 0.4–40 mg/L (for trigonelline) and 0.550 mg/L (for caffeine and chlorogenic acid) with an acceptable regression coefficient (*r* ^2^) of .9978, .9995, and .9995 for trigonelline, caffeine and chlorogenic acid respectively (Figures 2 and 3). The regression equation parameters of each analyte are summarized in Table 2.

**TABLE 2:** Linearity, accuracy, LOD, and LOQ parameters of the spectrophotometry method

Analyte	Slope	The standard error of the intercept	LOD (mg/L)	LOQ (mg/L)	*R* ^2^
Trigonelline	0.0328	0.01502	1.51	4.58	.9978
Caffeine	0.0458	0.01341	0.96	2.86	.99949
Chlorogenic acid	0.05604	0.01493	0.879	2.66	.99956

The LOD of the three analytes was found to be 1.51, 0.960, and 0.879 mg/L while the LOQ was 4.58, 2.86, and 2.66 mg/L, respectively, for trigonelline, caffeine, and chlorogenic acid (Table 2).

### 3.3 Determination of the three analytes in the green coffee extract

**FIGURE 4:**
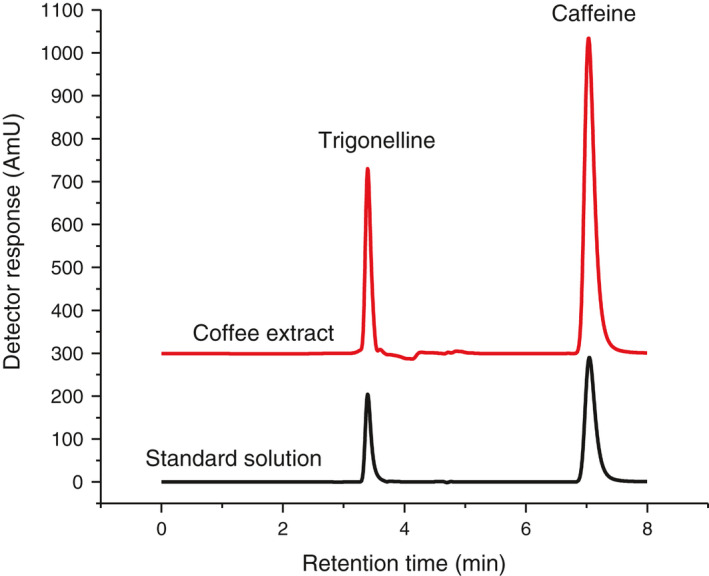
Representative HPLC chromatogram of coffee extract

The proposed UV‐VIS method was applied for the simultaneous determination of the three analytes in three green coffee samples. The obtained results for trigonelline and caffeine were compared with the results obtained by RP‐HPLC‐DAD. The obtained data are shown in Table 3 and a representative HPLC chromatogram is depicted in Figure 4. The concentration of trigonelline, caffeine, and chlorogenic acid in the green coffee samples, respectively, ranged 0.71%–1.04%w/w, 1.05%–1.21%w/w, and 8.50%–8.92%w/w. Comparable data were obtained with HPLC (Table 3) with correlation coefficient of 0.93 and 0.83, respectively, for trigonelline and caffeine. Furthermore, in order to evaluate the trueness or accuracy of the data obtained by the proposed method as compared to the reference HPLC‐DAD method, a two sided *t*‐test was applied (Boqué et al., 2002). Table 4 shows t‐calculated, F‐calculated, and the corresponding table values. Looking at Table 4, student *t*‐test analysis between the UV‐VIS and HPLC‐DAD did not show a significant difference at 95% confidence level. Therefore, the proposed method could provide an accurate data as that of the HPLC‐DAD method.

**TABLE 3:** Mean ± standard deviation (*n* = 3) of trigonelline, caffeine, and chlorogenic acid in three coffee samples obtained by UV‐VIS and HPLC methods

Coffee sample ID	UV‐VIS method (% wt/wt)						HPLC‐DAD method (wt/wt)			
	Trigonelline	%RSD	Caffeine	%RSD	Chlorogenic acid	%RSD	Trigonelline	%RSD	Caffeine	%RSD
A	0.71 ± 0.04	4.93	1.06 ± 0.08	7.36	8.50 ± 0.26	3.06	0.78 ± 0.07	8.33	1.01 ± 0.05	5.25
B	0.90 ± 0.05	5.56	1.05 ± 0.09	8.57	8.59 ± 0.59	6.87	0.87 ± 0.05	5.75	1.17 ± 0.08	6.84
C	1.04 ± 0.10	9.62	1.21 ± 0.11	9.09	8.92 ± 0.54	6.61	1.15 ± 0.17	14.78	1.31 ± 0.15	11.5

**TABLE 4:** Calculated and tabulated *t*‐and *F*‐values for the estimation of accuracy of the proposed method

Coffee sample ID	Trigonelline		Caffeine		F_tabulated_ for *α* = 0.05, v_1_ = 2 and v_2_ = 2	T_tabulated_ for *α* = 0.05 and 4 degree of freedom
	*F*_‐calculated_	*T*_‐calculated_	*F*_‐calculated_	*T*_‐calculated_		
A	3.45	1.34	1.47	0.75	19	2.78
B	1.00	0.60	1.13	1.41		
C	2.89	0.79	1.36	0.76		

In addition, the % RSD values for the three analytes determined by the proposed method and the reference method are ranged 3%–14% (Table 3, which indicates that the precision of the proposed method and the reference method were acceptable. The two‐sided *F*‐test also confirmed the absence of a significant difference between the UV‐VIS and HPLC‐DAD method for caffeine and trigonelline analysis (Table 4).

The caffeine and trigonelline results obtained in this study were in agreement with the data for Ethiopian coffee reported by Mehari, Redi‐Abshiro, Chandravanshi, Atlabachew, et al. (2016). On the other hand, the total chlorgenci acid content obtained in this study (8.50%–8.92%) were in agreement with the data reported by Scholz et al. (2016) (7.59%–10.11% wt/wt) and Tolessa et al. (2019) (5.41%–7.42% wt/wt) but slightly higher than the results reported by Mehari, Redi‐Abshiro, Chandravanshi, Combrinck, et al. (2016) (6.2%–6.8%). However Scholz et al. (2016) have quantified only the three major chlorogenic acids, while Mehari, Redi‐Abshiro, Chandravanshi, Combrinck, et al. (2016) have identified and quantified only 8 out of 18 separated chromatographic peaks. Thus, the slight variation between the current study and the reported data might be due to missing some of the unidentified minor chlorogenic acid by the reported methods.

Determination of the three coffee analytes based on salting‐out assisted liquid–liquid extraction followed by UV‐VIS analysis represented an alternative to gel filtration chromatography and RP‐HPLC. The former method is simple and inexpensive for the simultaneous determination of the three analytes.

## 4 CONCLUSION

The proposed method for the simultaneous determination of trigonelline, caffeine, and chlorogenic acid involves the use of QuEChERS salt and acetonitrile for the salting‐out assisted liquid‐liquid extraction followed by UV‐VIS determination at 264, 274, and 325 nm, respectively, for trigonelline, caffeine, and chlorogenic acid detections. The proposed method is useful for routine analysis of these compounds in various industries for quality control and traceability of the origin of the coffee samples.

## CONFLICT OF INTEREST

The authors declare that they do not have any conflict of interest.

## ETHICAL APPROVAL

This study does not involve any human or animal testing.

## Data Availability

The data that support the findings of this study are available from the corresponding author upon reasonable request.
